# Anti-Interferon Auto-Antibodies in Autoimmune Polyendocrinopathy Syndrome Type 1

**DOI:** 10.1371/journal.pmed.0030292

**Published:** 2006-06-13

**Authors:** Michael Levin

## Abstract

Levin discusses a new study, by Meager and colleagues, which identified a novel target of the disordered immune response in patients with APS-1.

Autoimmunity is a common mechanism underlying many common human diseases. Although the mechanisms are not well understood, autoimmunity is thought to arise from a failure in self-tolerance, resulting in a sustained immunological attack by specific antibody, T cells, or both, directed against antigens within the target tissues or organs [ 1]. Many autoimmune disorders appear to have a genetic basis, but attempts to identify the human genes involved have had only limited success, probably because of the polygenic nature of most common autoimmune disorders, and because of the complexity of the immunological pathways involved [ 2].

Many of the most important clues as to the working of the human immune system have come from the study of patients with rare single gene defects. Autoimmune Polyendocrinopathy Syndrome Type 1 (APS-1) is a rare, recessively inherited disorder, which is more common in the Finnish, Sardinian, and Iranian Jewish populations than in the general population[ 3]. The disorder usually presents in early childhood, with chronic mucocutaneous candidiasis, and adrenal or para-thyroid failure. The clinical manifestations of the disorder are extremely variable and include diabetes, keratitis, chronic diarrhoea, alopecia, hepatitis, pernicious anaemia, and primary hypogonadism [ 4].

Immunologically, the disease is characterised by lymphocytic infiltration of the target organs and by the presence of auto-antibodies against a wide range of tissue-specific antigens [ 5, 6]. Mucocutaneous candidiasis occurs in all patients with APS-1, but the immunological basis of the failure to eliminate candida is not understood. In general, the defect in elimination of candida is not associated with defective handling of other pathogens, suggesting a candida-specific immune defect [ 7].

## The Molecular Basis of Type 1 APS

A breakthrough in understanding the molecular basis of Type 1 APS came from identification of the causative gene by positional cloning [ 8–10]. The novel gene, named *AIRE* (autoimmune regulator), codes for a 545–amino acid protein that acts as a transcriptional regulator and which probably plays a role in regulating self-antigen expression in medullary thymic epithelial cells and dendritic cells [ 11, 12]. Different mutations in the coding region of the *AIRE* gene are responsible for APS-1 in patients carrying homozygous or multiple heterozygous mutations [ 13].

Although the *AIRE* gene defines novel pathways controlling self-tolerance, many questions about the immunopathogenesis have remained unanswered: 1) the disease is extremely variable in its clinical and immunological manifestations even in patients carrying the same mutation; 2) the relationship between *AIRE* mutations and impaired immunity to candida remains unknown; and 3) how the AIRE protein controls self-tolerance remains poorly understood.

## A New Study Shows Antibodies against Interferons

Based on the observation that chronic mucocutaneous candidiasis is also seen in patients with thymoma and myasthenia gravis, in whom high titres of antibodies against interferon alpha and IL-12 have been found [ 14], Meager and colleagues speculated that a similar pathogenic mechanism might be involved in patients with APS-1. They studied two well-characterised cohorts of Finnish and Norwegian APS-1 patients, and they reported their results in *PLoS Medicine* [ 15]. Using both ELISA-based assays and functional interferon neutralising assays, they documented high titre IgG auto-antibodies against Type 1 interferons in 100% of patients with APS-1, but not in healthy controls, in heterozygous carriers of the *AIRE* mutations, or in people with other endocrine disorders.

The anti-interferon antibodies neutralised the biological activity of interferon alpha and interferon omega, as well as the activity of mixed interferons produced in virally stimulated cells. The anti-interferon antibodies occurred prior to the development of other auto-antibodies, and in some patients preceded the development of clinical features of APS-1.

Meager and colleagues' study has identified a novel target of the disordered immune response in patients with APS-1, and suggests a role for Type 1 interferons in immune responses to candida and in the regulation of self-tolerance. The findings may have both clinical relevance and implications for the understanding of the immunological events in autoimmunity. The finding of anti-interferon antibodies early in the course of the disease in 100% of patients carrying *AIRE* mutations suggests that these antibodies may serve as a diagnostic marker of patients carrying homozygous *AIRE* mutations. These antibodies may therefore be useful for screening family members or patients with only some features of the disorder. The consistent prevalence of anti-interferon antibodies in all patients with APS from the time of presentation suggests that the development of these antibodies has been inherited concurrently with the *AIRE* mutations, behaving as a recessive genetic trait with complete penetrance. This raises a number of questions about the role of these antibodies in the later manifestations of the disease.

## Implications of the Study

Why should anti-interferon antibodies develop with such consistency in patients with homozygous *AIRE* mutations, and what is their significance in the immunopathogenesis of the disorder? Impairment of the anti-infective properties of Type 1 interferons might be expected to result in infection with a wide range of opportunistic pathogens. However, apart from candida, severe infections with other pathogens are rare in this disorder. This may suggest redundancy in the requirement for interferons for most infections (with other cytokines compensating for the lack of effect of Type 1 interferons). Alternatively, Type1 interferons may have a specific role in immunity to candida not shared by other pathogens. Such specificity in requirement for individual cytokines in immunity is seen in the case of patients with defects in the interferon gamma and IL-12 pathways, who are highly susceptible to mycobacteria and intracellular pathogens while manifesting normal immunity to most other organisms [ 16, 17].

As with many important novel observations, Meager and colleagues' report raises more questions than it answers, and should stimulate research to unravel the role of Type 1 interferons and their antibodies in self-tolerance and autoimmunity. Does the early appearance of anti-interferon antibodies suggest a key role for interferons in elimination of autoreactive T and B cell responses? If so, does the impaired interferon response play a role in the relentless T cell and antibody attack on the other tissues and organs occurring in this disorder?

The definitive observation made in this report was only possible because clinical investigators in several countries had assembled large cohorts of patients with a rare genetic disorder, and had collected clinical samples and patient information over a period of several decades. At a time when it is difficult to obtain research funding or even ethical approval for long term, open-ended clinical research, this study is a welcome reminder of the importance of long-term follow-up of patients with rare diseases. Such patients continue to provide unique human models through which to gain insights into the complex workings of the human immune system. While the anti-interferon antibodies may have immediate clinical use in diagnosis of APS-1, unravelling of the mechanisms involved in their production, and the immunological consequences of their presence in the circulation, should be a fruitful area for research to understand autoimmunity.

## Abbreviations

*AIRE*: autoimmune regulator
APS-1: Autoimmune Polyendocrinopathy Syndrome Type 1
